# A stochastic version of the Price equation reveals the interplay of deterministic and stochastic processes in evolution

**DOI:** 10.1186/1471-2148-8-262

**Published:** 2008-09-25

**Authors:** Sean H Rice

**Affiliations:** 1 Department of Biological Sciences, Texas Tech University, Lubbock, TX 79409, USA

## Abstract

**Background:**

Evolution involves both deterministic and random processes, both of which are known to contribute to directional evolutionary change. A number of studies have shown that when fitness is treated as a random variable, meaning that each individual has a distribution of possible fitness values, then both the mean and variance of individual fitness distributions contribute to directional evolution. Unfortunately the most general mathematical description of evolution that we have, the Price equation, is derived under the assumption that both fitness and offspring phenotype are fixed values that are known exactly. The Price equation is thus poorly equipped to study an important class of evolutionary processes.

**Results:**

I present a general equation for directional evolutionary change that incorporates both deterministic and stochastic processes and applies to any evolving system. This is essentially a stochastic version of the Price equation, but it is derived independently and contains terms with no analog in Price's formulation. This equation shows that the effects of selection are actually amplified by random variation in fitness. It also generalizes the known tendency of populations to be pulled towards phenotypes with minimum variance in fitness, and shows that this is matched by a tendency to be pulled towards phenotypes with maximum positive asymmetry in fitness. This equation also contains a term, having no analog in the Price equation, that captures cases in which the fitness of parents has a direct effect on the phenotype of their offspring.

**Conclusion:**

Directional evolution is influenced by the entire distribution of individual fitness, not just the mean and variance. Though all moments of individuals' fitness distributions contribute to evolutionary change, the ways that they do so follow some general rules. These rules are invisible to the Price equation because it describes evolution retrospectively. An equally general prospective evolution equation compliments the Price equation and shows that the influence of stochastic processes on directional evolution is more diverse than has generally been recognized.

## Background

Evolution involves both deterministic processes, such as selection, and random processes such as drift. When deterministic and stochastic processes are combined in the same model it is common to use the "diffusion approximation" – essentially assuming that populations are large (so that evolution can be approximated as a continuous process), that population size is relatively stable, and that selection is weak [1-4]. The diffusion approximation is nearly always used when analytical (rather than numerical) solutions are sought.

The diffusion approximation has yielded many important results concerning the interaction of deterministic and stochastic evolutionary processes. In particular, a number of different models have shown that the direction of evolution is influenced not only by the relative mean fitnesses of different strategies (or alleles) but also by the variances in possible fitness values associated with each strategy [5-10]. If the variance in each individual's fitness distribution influences directional evolution, then it seems likely that other aspects of the fitness distribution (*i.e*. other moments) should do so as well. However, most of the models that have been studied have used methods (such as the Itô calculus [11]) which make it difficult to see the effects of higher moments of the fitness distribution of an individual.

The most general (in the sense of making the fewest simplifying assumptions) mathematical description of evolution that we currently have, the Price equation [12], does not easily accommodate stochastic evolutionary processes. The Price equation is an exact description of the relation that must hold between the phenotype of parents, the fitness of parents, the difference between parents and offspring, and evolutionary change [13]. Unfortunately, all of these parameters must be specified exactly. The Price equation is thus exact only in hindsight, after reproduction has taken place and we know the precise value of each individual's fitness and the mean phenotype of its offspring.

Despite this apparent limitation, the Price equation has been used extensively to study social evolution [14-16], the foundations of quantitative genetics [13,17], and the analysis of multilevel selection [13,18-20] as well as in other fields such as ecology [21,22]. Since all of these fields also involve stochastic processes, it would be of value to have a theory with the generality of the Price equation that does not require that all parameters are known exactly to begin with.

Below, I present a general equation for directional evolutionary change that treats fitness and offspring phenotype as random variables, rather than numbers, but imposes no restrictions on the distributions associated with these random variables. This is essentially a stochastic version of the Price equation, though it is derived independently and contains a term not found in Price's formulation. This theory accommodates all processes that influence directional evolution, both deterministic and stochastic. Using this result, I show that deterministic and stochastic processes interact in complex ways. One result is that stochastic variation in fitness amplifies the effects of selection in small or fluctuating populations. Furthermore, the role of fitness variation within an individual is more complex than has generally been recognized. The well known tendency for populations to be pulled towards phenotypes with minimum variance in fitness turns out to be one instance of a more general rule that, all else held equal, populations are pulled towards phenotypes with minimum symmetrical variation in fitness, as measured by all of the even moments of an individual's fitness distribution. This process can actually cause the variance in fitness to increase (so long as higher even moments decrease). There is also a tendency for populations to be pulled towards phenotypes with maximum positive asymmetry in fitness (as measured by the odd moments). Finally, this equation contains a term, capturing the direct effects of reproduction on offspring phenotype, that has no analog in the Price equation.

## Results

In the following analysis, the fitness of an individual (*w*) measures the number of descendants that the individual has at some future time, potentially including the individual itself [13]. We consider a population of individuals that have not yet reproduced, and therefore treat fitness and offspring phenotype not as fixed values, but as random variables, each having a distribution of possible fitness values. The mean of an individual's fitness distribution, $\hat{w}$, is the number of descendants that the individual is expected to leave.

Because each individual has a distribution of possible fitness values, the mean fitness in the population ($\bar{w}$), which determines population growth rate, is also a random variable. If $\bar{w}$ = 0 then the population goes extinct, and the change in mean phenotype is undefined. We thus define $\Omega =\left(\frac{w}{\bar{w}}|\bar{w}\neq 0\right)$ as the ratio of individual fitness to mean population fitness, conditional on the population not going extinct. Throughout this discussion, $\bar{x}$ refers to the average value of *x *in a population, and $\hat{x}$ refers to the expected value of random variable *x*.

### The general equation

Using the notation given in Table 1, the expected change in mean phenotype over some interval (denoted $\hat{\Delta \bar{\phi }}$) is given by (see Methods for derivation):

**Table 1:** Symbols and notation.

Symbol	Meaning
*N*	Population size
*ϕ*	Phenotype of an individual
*ϕ*^*o*^	Mean phenotype of an individual's offspring
*δ*	*ϕ*^*o *^- *ϕ*
$\hat{\bar{\delta }}$	Expected mean value of *δ *in the population
*w*	Fitness of an individual
$\tilde{w}$	Expected fitness in the current environment
*f*_*ϕ*_	Frequency of phenotype *ϕ *in the population
Ω	$\frac{w}{\bar{w}}$ conditional on $\bar{w}$ ≠ 0
H($\bar{w}$)	Harmonic mean of $\bar{w}$
μ_*i*_(*w*)	*i*^*th *^central moment of *w*
$\bar{X}$ or Ave (*X*)	Average value of *X *in population
$\hat{X}$ or E(*x*)	Expected value of random variable *X*

(1) $$\hat{\Delta \bar{\phi }}=\mathrm{cov}(\hat{\phi^{o}},\hat{\Omega })+\bar{{\mathrm{cov}}_{i}(\phi^{o},\Omega )}+\hat{\bar{\delta }}$$

This is essentially a stochastic version of Price's theorem. Note, though, that it contains a term that has no analog in Price's formulation. This new term, $\bar{{\mathrm{cov}}_{i}(\phi^{o},\Omega )}$, describes the population average of the covariance, within an individual, between the average phenotype of that individual's offspring (*ϕ*^*o*^) and the individual's contribution to population growth (Ω). This term does not appear in Price's theorem because that equation treats offspring phenotype and fitness as parameters, rather than random variables (*i.e*. each individual has a specific value of *ϕ*^*o *^and of fitness, rather than a distribution of possible values for each of these). This is why Price's theorem is exact only after reproduction has taken place.

We can write Equation 1 in more familiar form by defining *δ *as the difference between the mean phenotype of an individual's offspring and that individual's own phenotype, then substituting *ϕ *+ *δ *for *ϕ*^*o*^, to yield:

(2) $$\hat{\Delta \bar{\phi }}=\mathrm{cov}(\phi ,\hat{\Omega })+\mathrm{cov}(\hat{\delta },\hat{\Omega })+\bar{{\mathrm{cov}}_{i}(\delta ,\Omega )}+\hat{\bar{\delta }}$$

Note that *ϕ*, the current phenotype of an individual, is not treated as a random variable. This is because, at whatever time we look at the system, *ϕ *already has a value for each individual. By contrast, *w *and *δ *are random variables because they concern future events and thus could have a range of possible values. Terms in Equation 2 containing *δ *concern processes, such as mutation and recombination, that cause offspring to, on average, differ from their parents. If we set *δ *= 0, we are left with only cov(*ϕ*,$\hat{\Omega }$), which is the change due only to differential survival and reproduction. This term corresponds to the "selection differential" term in the Price equation [13]. However, we will see that because both individual fitness (*w*) and mean population fitness ($\bar{w}$) are now random variables, the term cov($\hat{\phi },\hat{\Omega }$) now contains more than just selection.

Because it is the expected value of the ratio of two correlated random variables, ${\hat{\Omega }}_{k}$ (the expected value of the ratio of individual *k*'s fitness to mean population fitness conditional on $\bar{w}$ ≠ 0) can behave in unexpected ways. In order to tease these apart, we can expand ${\hat{\Omega }}_{k}$ to yield (see Methods):

(3) $${\hat{\Omega }}_{k}=\frac{{\hat{w}}_{k}}{\text{H}(\bar{w})}+\sum_{i=1}^{\infty }\frac{{\left(-1\right)}^{i}\mu_{i+1}(w_{k}{\bar{w}}^{i})}{{\hat{\bar{w}}}^{i+1}}$$

Here, H($\bar{w}$) is the harmonic mean of the distribution of possible values of $\bar{w}$, and μ_*i*+1 _(*w*_*k *_${\bar{w}}^{i}$) is the (*i *+ 1)^*st *^mixed central moment of *w*_*k *_and ${\bar{w}}^{i}$. (The first of these terms, μ_2_(*w*_*k *_$\bar{w}$), is the covariance between individual fitness and mean population fitness). The value of the μ_*i*+1_(*w*_*k *_${\bar{w}}^{i}$) terms is determined by the source of random variation in fitness. We will consider two special cases: pure demographic stochasticity and random environmental change. For this discussion, we will set *δ *= 0, which is equivalent to looking only at the "selection differential", *S*, which ignores mutation, recombination, and other processes that could cause offspring to not resemble their parents.

### Demographic stochasticity in a constant environment

Even in an environment that seems constant to an outside observer, there will be variation in individual fitness values, even among individuals with the same phenotype. This variation corresponds to what is generally called demographic stochasticity, and it will be present in all populations [23]. Pure demographic stochasticity is roughly equivalent to the "within-generation" component of variation discussed by Gillespie [7].

If the fitness values of different individuals are independent (meaning that the number of descendants of individual *j *is independent of whether individual *i *leaves more or fewer descendants than expected), and the environment does not change from generation to generation, then we can find the selection differential (*S*) by expanding cov(*ϕ*, $\hat{\Omega }$). Considering only the first three terms in the expansion, this yields:

(4) $$\hat{S}\approx \frac{\mathrm{cov}(\phi ,\hat{w})}{\text{H}(\bar{w})}-\frac{\mathrm{cov}(\phi ,\mathrm{var}(w))}{N{\hat{\bar{w}}}^{2}}+\frac{\mathrm{cov}(\phi ,\mu_{3}(w))}{N^{2}{\hat{\bar{w}}}^{3}}$$

Here, *N *designates actual, rather than effective, population size. The three terms on the right-hand side of Equation 4 each correspond to different directional evolutionary forces acting on the population. These are: 1) selection (here a function of *N *because of the H($\bar{w}$) term), 2) a force pulling the population towards phenotypes with minimum variance in fitness, and 3) a force pulling the population towards phenotypes with maximum positive skewness in fitness. The terms corresponding to higher moments of the distribution of fitness follow the same pattern; those containing even central moments are negative and those containing odd central moments are positive.

### Random environmental change

In addition to pure demographic stochasticity, the environment may change over time in ways that differentially affect different phenotypes (the "between-generation" component of Gillespie [7]). In this case, the expected fitness of individuals with a particular phenotype will itself vary over time, so the total fitness distribution of an individual will be a function of both the distribution of expected fitness values, given its phenotype, and the distribution of variation around this expected value due to demographic stochasticity. In such a case, we can write the fitness of individual *i *as *w*_*i *_= ${\tilde{w}}_{i}$ + *s*_*i*_, where ${\tilde{w}}_{i}$ is the expected fitness in the current environment of individuals with the same phenotype as *i*, and *s*_*i *_is the deviation of individual *i *from this expectation due to pure demographic stochasticity.

If we denote the frequency of phenotype *ϕ *in the population as *f*_*ϕ*_, then in a very large population, the expected change in mean phenotype is approximated (to the first three terms) by:

(5) $$\hat{S}\approx \frac{\mathrm{cov}(\phi ,\hat{w})}{\text{H}(\bar{w})}-\frac{\mathrm{cov}(\phi ,f_{\phi }\mathrm{var}(\tilde{w}))}{{\hat{\bar{w}}}^{2}}+\frac{\mathrm{cov}(\phi ,f_{\phi }^{2}\mu_{3}(\tilde{w}))}{{\hat{\bar{w}}}^{3}}$$

Equations 4 and 5 have the same form. The difference is that in Equation 4 we are assuming that the fitness of each individual is independent of the fitness of every other individual, whereas in Equation 5 we assume that the fitness values of all individuals with the same phenotype are correlated, since they are all influenced in the same way by the environment. For intermediate sized populations experiencing a varying environment, both var(*s*) and var($\tilde{w}$) will enter the calculations (see Methods).

## Discussion

Equations 1 and 2 apply to any evolving system. These equations are based only on the assumption of a population of things that leave descendants and have measurable phenotypes, and they encompasses all factors, both deterministic and stochastic, that contribute to directional evolutionary change in a closed population. If we specify the exact population size in the next generation (fixing the value of $\bar{w}$), and fix the value of *δ *for each individual, then Equation 2 becomes equivalent to the Price equation with fitness simply replaced by expected fitness, $\hat{w}$[24]. For simplicity, I will often refer to ancestors as "parents" and descendants as "offspring", with the understanding that the same equation applies regardless of the time interval over which we look. Furthermore, the ancestors and descendants need not be the same type of biological unit. For sexually reproducing organisms, we can treat a mated pair as the ancestor and an individual offspring as a descendant, or an individual as the ancestor and a successful gamete as the descendant. Descendants may also include the ancestors at a later time, allowing for overlapping generations.

The phenotype, *ϕ*, may be any measurable trait. This fact allows us to derive much of classical evolutionary theory from Equation 2 simply by choosing the appropriate phenotype. For example, we can derive standard population genetic models for change in frequency of an allele, *A*, by defining the phenotype (*ϕ*) of an individual as the frequency of *A *within that individual's genotype (*ϕ *is therefore 0, 0.5, or 1). Defining *ϕ *in this way, $\bar{\phi }$ is equal to the frequency of the *A *allele in the population [13,25], so Equation 2 gives the change in allele frequency.

Many (though not all) of the evolutionary processes that I discuss in the following sections appear because $\bar{w}$ is a random variable. This is a biologically interesting case because demographic stochasticity – stochastic fluctuations in natural populations due to variation in individual reproduction – is ubiquitous in nature [23]. In most of the following discussion, I will focus on the special case in which the fitness values of different individuals within the same generation are independent. It is important to note that this does not preclude density dependent population regulation. For example, if all individuals in a population happen to produce more offspring than needed for replacement, then the population size will increase. In the next generation, though, the resulting increased competition may reduce the fitness of all descendants, preventing (or reducing the probability of) further population increase. If this reduced fitness of descendants is manifest as reduced viability, then this is equivalent to the different "culling" processes discussed by Gillespie [26]. In the case of "exact culling" [9], the mean phenotype is unchanged by the culling process, so the changes in mean phenotype discussed here will occur even though the population does not increase over multiple generations.

It is of course possible for density dependence to involve a direct influence of one individual's reproduction on that of another. One example is the case of cavity nesting birds where the number of suitable cavities is fixed. In this case, the act of one pair locating a cavity directly reduces the probability that another pair will do so. In such cases, there will be a negative covariance between the fitness values of different individuals (or pairs). This negative covariance will appear in the values of the μ_*i*+1_(*w *${\bar{w}}^{i}$) terms in Equation 3. Specifically, making no assumptions about independence of fitness values, $\mu_{2}(w_{i}\bar{w})=\frac{1}{N}\mathrm{var}(w_{i})+\frac{N-1}{N}\bar{\mathrm{cov}(w_{i},w_{j\neq i})}$. The term $\bar{\mathrm{cov}(w_{i},w_{j\neq i})}$ is the average covariance between individual *i*'s fitness and that of other members of the population.

The first term on the righthand side of Equation 2, cov(*ϕ*, $\hat{\Omega }$), captures the contribution of differential survival and reproduction to directional evolutionary change. Though this is traditionally called the "selection differential" [13], the expansion of this term (Equations 4 and 5) shows that stochastic processes can contribute substantially to directional evolution, both in small populations and in populations subjected to random environmental variation. In this discussion, I will define "selection" as differential expected production of descendants that is causally determined by differences in phenotype. Under this definition, some of the processes that contribute to cov(*ϕ*, $\hat{\Omega }$) are not kinds of selection. I will nonetheless continue to use "selection differential", designated *S*, because it is the standard term.

Equations 4 and 5 show the expected selection differential for cases corresponding to different sources of fitness variation. The difference between these equations makes sense when we note that, in Equation 5, all individuals with the same phenotypic value have the same fitness in any particular generation. What matters is thus the frequencies of the different phenotypic values (*f*_*ϕ*_). This is also true in Equation 4. Here, however, each individual's fitness is independent of that of all other individuals, so each individual is effectively its own "type", with frequency $\frac{1}{N}$. It thus makes sense that the powers of $\frac{1}{N}$ in Equation 4 are replaced, in Equation 5 by powers of *f*_*ϕ*_.

### The expected selection differential is amplified by random variation in fitness

The first term on the righthand side of Equation 4, cov(*ϕ*, $\hat{w}$)/H($\bar{w}$), shows that the magnitude of the expected selection differential increases with increasing variation in $\bar{w}$. This follows from the fact that the term cov(*ϕ*, $\hat{w}$) which captures the effects of selection, is divided by the harmonic mean of $\bar{w}$, H($\bar{w}$). Since the harmonic mean is disproportionately influenced by small values, H($\bar{w}$) will tend to decrease as the variation in $\bar{w}$ increases, as is expected in small populations or in a variable environment. Equation 17 in the Methods section shows how 1/H($\bar{w}$) depends on variation in $\bar{w}$.

**Figure 1:**
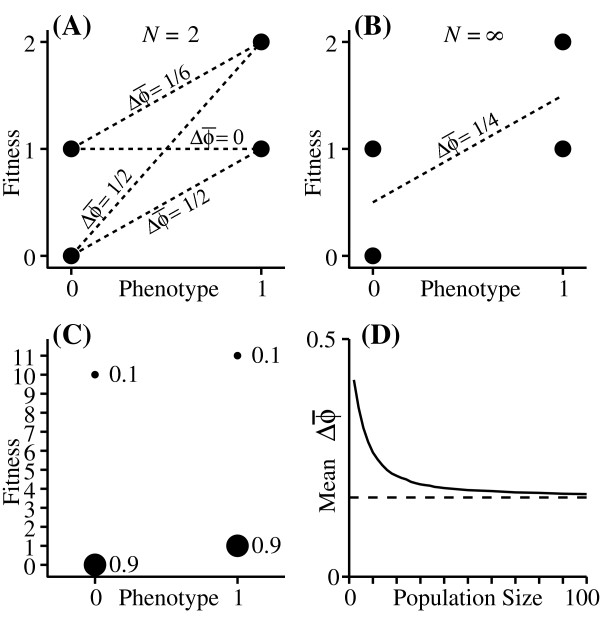
**Amplification of expected selection differentials in small populations**. Fitness distributions for two phenotypes; the size of the dot indicates the probability of that fitness value. In (A) and (B), Individuals with phenotypic value 0 leave either 0 or 1 descendant with equal probability, and those with phenotype 1 leave either 1 or 2 descendants with equal probability. In (A), there is one individual with each phenotype. The lines show the four possible (and equally probable) combinations of fitness values, with the corresponding Δ$\bar{\phi }$. The average change is E(Δ*ϕ*) = $\frac{7}{24}$. (B): In an infinite population evenly divided between the two phenotypes, the total contribution of individuals with phenotype *ϕ *= 1 will always be 3 times greater than the total contribution of individuals with *ϕ *= 0, yielding $\text{E}(\Delta \bar{\phi })=\frac{1}{4}=\frac{6}{24}$, which is the prediction of classical theory. (C): Another example of fitness distributions leading to directional selection. Numbers adjacent to dots are probabilities. (D): Results of monte-carlo simulations using the fitness distributions in (C). The dashed line is the value for *N *= ∞.

To understand the biology behind this phenomenon, note that the selection differential is inversely proportional to mean population fitness ($\bar{w}$); it is thus disproportionately influenced by small values of $\bar{w}$ (Fig. 1A). For a population of size *N*, $\bar{w}$ is essentially the mean of a sample of *N *points drawn from the overall fitness distribution. In a very large population (*i.e*. a very large sample), the value of $\bar{w}$ will nearly always be very close to the expected value, $\hat{\bar{w}}$. By contrast, in a small population, there is a significant chance that $\bar{w}$ will be much larger or much smaller than $\hat{\bar{w}}$. Since the small values have a disproportionate effect on the selection differential, the expected selection differential increases as population size decreases. The same thing occurs even in large populations if $\bar{w}$ is uncertain due to random environmental fluctuations. In order to test this conclusion, I performed monte-carlo simulations, following a population over one generation, using the fitness distributions in Fig. 1C. The mean change in $\bar{\phi }$, averaged over 100,000 runs, is shown in Fig. 1D. Note that in this case, the expected change due to selection in a very small population can be substantially larger than would be expected from classical theory. In this example, the environment is held constant, so the amplification of the selection differential decays with increasing population size. If the variation in $\bar{w}$ is a consequence of environmental variation that differentially affects different phenotypes, then we will see the same amplification in large populations as well. The "Worked example" section in Methods explains how to calculate 1/H($\bar{w}$) from the individual fitness distributions.

Though this phenomenon is not generally recognized in the literature, a special case can actually be derived from equations in Gillespie's 1977 paper [27] and in Proulx [9]. (In Equation 7 in [27], set var(*X*) = var(*Y*) and $\bar{X}\neq \bar{Y}$, using the notation of that paper. I am indebted to Steve Proulx for pointing this out). In this special case, the expected change increases with the variance in individual fitness values. In general, Equations 3 and 17 show that all of the moments of the individual fitness distributions contribute to 1/H($\bar{w}$), and Figure 5 shows that considering only the variance can easily underestimate the degree to which the effects of selection are amplified.

This phenomenon at first seems at odds with the theoretical [28] and experimental [29] studies that have suggested that the average long term response to selection increases with increasing *N*, resulting from the increased availability of genetic variation in larger populations [29]. The reason that this effect has been missed is that theoretical studies have treated $\bar{w}$ as a fixed parameter (or, equivalently, they hold population size fixed, as in Robertson's theory of selection limits [28]). Holding $\bar{w}$ fixed means that $\hat{\Omega }=\frac{\hat{w}}{\bar{w}}$, which is independent of population size (compare with Equation 14, in which $\bar{w}$ is not fixed). Experimental studies have effectively done the same thing, by choosing the same number of individuals in each round of selection and by using truncation selection [29], minimizing the variation in individual fitness. Recent theoretical and empirical studies concerning the adaptive potential of small populations [30,31] have considered the effects of population size only on genetic variation, assuming that the selection differential is independent of population size. The loss of heritable variation should indeed cause the long term response to selection to be reduced in small populations. Over the short term, though, the amplification described here should facilitate a rapid adaptive response over the first few generations. Such an amplified selection response could contribute to population differentiation in peripheral isolates.

### The even-moment effect: Populations are pulled towards phenotypes having minimum symmetrical variation in fitness

Symmetrical spread about the mean of a distribution is measured by the even central moments. In the summation on the right-hand side of Equation 3, the terms containing even moments are all negative (since, if *i *+ 1 is even, *i *is odd so (*-*1)^*i *^= *-*1). The covariance between phenotype and these terms thus corresponds to the population being pulled towards phenotypes with minimal symmetrical variation. This is apparent in Equations 4 and 5, in which the term containing the variance (the second moment) is negative.

**Figure 2:**
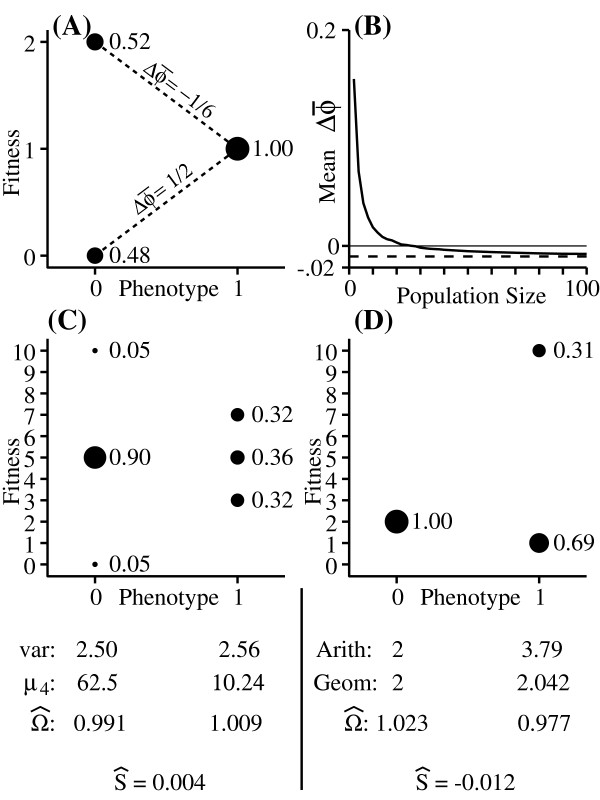
**The even-moment effect**. (A): Case in which individuals with phenotype 0 have a higher expected fitness than do those with phenotype 1 (${\hat{w}}_{0}$ = 1.04, ${\hat{w}}_{1}$ = 1) as well as a higher variance in fitness (var(*w*_0_) = 0.9984, var(*w*_1_) = 0). (B): The mean value of Δ$\bar{\phi }$ from monte-carlo simulations, given the fitness distributions in (A) and *δ *= 0. (C): Case in which both phenotypes have the same mean fitness, and the expected direction of evolution is towards the phenotype with the higher variance in fitness (but the lower fourth moment). The variance, fourth moment, and $\hat{\Omega }$ for each phenotype are shown below the distributions (D): Example of a case in which, for *N *= 2, the expected direction of evolution is towards the phenotype with lower geometric mean fitness.

This is illustrated in Figs. 2A and 2B. The even-moment effect results from the fact that the fitness of individuals (or phenotypes) with the most variable fitness covary most strongly with $\bar{w}$[32]. When those individuals with high variation in fitness leave many descendants, the value of $\bar{w}$ also tends to be high, reducing the magnitude of change. Conversely, when those with high variation leave few descendants (and therefore decrease in frequency), $\bar{w}$ tends to be low, increasing the magnitude of the decline (Fig. 2A). In a constant environment, this effect drops off with increasing population size, since the even moments of $\bar{w}$ are all divided by increasing powers of 1/*N*. As with the amplification of selection differentials discussed above, though, the even-moment effect remains strong in large populations when variation in individual fitness is due largely to environmental variation.

The tendency of populations to be pulled towards phenotypes with low variance in fitness has been noted by many authors [5,6,8,9,32-34]. Most of these studies used some form of the diffusion approximation, and thus assumed that higher moments could be ignored (though Proulx [9] presents an equation that can be expanded to yield the effects of higher moments, and notes that these need to be considered when the variation in fitness for each individual is not small). Equation 3 shows that, in fact, all even moments contribute to this phenomenon. To illustrate this, Figure 2C shows a case in which the expected direction of evolution is towards the phenotype with the higher variance in fitness. The reason is that the fourth and higher even moments of the fitness distribution associated with the phenotype with higher variance are much smaller than those associated with the other phenotype. If the variation in fitness is due to pure demographic stochasticity alone, then in this example variance in fitness is expected to increase only in very small populations, since the fourth moment term will be divided by *N*^3 ^and so will drop off quickly as *N *increases. On the other hand, if variation in fitness is primarily a result of environmental variation, then the fourth moment term will be multiplied by $f_{\phi }^{3}$ rather than $\frac{1}{N^{3}}$, so the higher moments may have an influence even in large populations, especially when the different phenotypes have similar frequencies.

The even-moment effect has sometimes been associated with the idea that selection acts on the geometric mean of individuals' fitness distributions. [35,36]. While geometric mean fitness is appropriate when fitness varies in a deterministic and predictable manner over time, it is not relevant in the case discussed here, where fitness is a random variable within a generation [24,27,32]. To illustrate this, Fig. 2D shows a case in which the strategy with the lowest geometric mean fitness is the one that is expected to increase in frequency. Instead, the direction of evolution is determined by $\hat{\Omega }$. Specifically, when ${\hat{\Omega }}_{i}$ > 1, the descendants of individual *i *are expected to comprise an increasing proportion of the population. Though $\hat{\Omega }$ resembles traditional "relative fitness", the fact that $\bar{w}$ is a random variable that is correlated with *w *means that $\hat{\Omega }$ does not scale like relative fitness (which preserves the relative order of the fitness values of different individuals [37]). This is why it is possible to have ${\hat{\Omega }}_{0}<{\hat{\Omega }}_{1}$ even if ${\hat{w}}_{0}$ > ${\hat{w}}_{1}$, in which case the trait that is expected to increase in frequency is also the one that causes individuals possessing it to have the lowest expected reproductive output (Fig. 2A). The "expected relative fitness" discussed by Lande [32] is a special case of $\hat{\Omega }$ (See Methods). Note that the term "relative fitness" is used in different ways in the literature. In some cases, relative fitness refers to the fitness of an individual (or a phenotype) divided by mean population fitness (*i.e*. $\frac{w_{i}}{\bar{w}}$) [10,24]. In other cases, relative fitness refers to the fitness of one individual or phenotype divided by the fitness of another individual or phenotype (this is the interpretation that suggests the importance of geometric mean fitness [38,39]). The exact reason that these two interpretations yield different results will be discussed elsewhere. For now, Fig. 2D is sufficient to show that geometric mean fitness does not necessarily identify which strategy will increase. Though the even moment effect is sometimes referred to as selection acting on variance, I argue below that the even-moment effect should not be treated as a kind of selection.

Previous discussions of the even-moment effect have treated it as a function only of population size. However, Equation 4 shows that this effect scales as 1/(${\hat{\bar{w}}}^{2}$); it is thus amplified if $\hat{\bar{w}}$ < 1, meaning that the population is expected to decline in size. The pull towards phenotypes with minimum variance in fitness can thus be important even in larger populations if they are rapidly declining. The degree to which declining population size amplifies the even-moment effect will depend on how the variance (and higher even moments) scales with the mean. In the extreme case in which the variance in fitness is independent of the mean, declining populations will be strongly influenced by the even-moment effect. As an example, consider a population of 10,000 individuals that is declining such that the expected number of individuals in the next time interval is 1000. In this case, The strength of the force pulling the population towards phenotypes with minimum variance in fitness is the same as it would be in a stable population with the same variances in fitness and size *N *= 100. (since, if *N *= 10,000 and $\hat{\bar{w}}$ = 0.1, *N *${\hat{\bar{w}}}^{2}$ = 100.) If the fitness distributions are approximately Poisson, then the variance will scale linearly with the mean and so dividing by ${\hat{\bar{w}}}^{2}$ will still amplify the even-moment effect, though to a lesser degree.

The fact that declining populations may be particularly prone to the even-moment effect could have consequences for the probability of extinction. Stochastic extinction – resulting from chance fluctuations in population growth rate – is a substantial threat to very small populations [40,41]. If a declining population shifts towards phenotypes that have minimum variance in fitness, then this could reduce the chance of stochastic extinction when the population becomes very small. Further study will be necessary to determine if this phenomenon can significantly influence extinction probabilities.

### The odd-moment effect: Populations are pulled towards phenotypes with maximum positive asymmetry of fitness

This follows from the fact that the odd moment terms on the right-hand side of Equation 3, which measure asymmetry of the fitness distribution, are all positive. Real fitness distributions will almost always be asymmetrical. This follows from the fact that individual fitness can not be less than zero but could possibly be very large, and that $\hat{w}$ will usually be close to 1.

In the case of pure demographic stochasticity, the odd-moment effect will be noticeable only in very small populations, since the third moment term in Equation 3 is divided by *N*^2^, the fifth moment term by *N*^4^, and so on. As with the even-moment effect discussed above, the odd-moment effect may be significant even in large populations when fitness variation is due to environmental fluctuations. For example, a phenotype that normally has moderate fitness but does much better than others during rare good years may show a long term increase that is greater than would be expected from the mean and variance of its fitness distribution.

**Figure 3:**
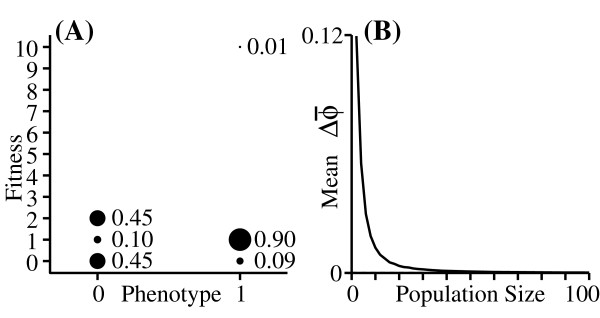
**The odd-moment effect**. (A): Fitness distributions for two phenotypes that have the same means ($\bar{w}$ = 1) and variances (var(*w*) = 0.9) but different third moments. (B): Simulation results for the change in mean phenotype given the fitness distributions in (A).

Note that the asymmetry that we are considering here is in the distribution of possible fitness values of an individual (*e.g*. the distribution associated with *ϕ *= 1 in Figure 3A). This is quite different from the "asymmetric fitness function" often discussed in the evolutionary genetics literature [25,42], which describes a case in which the plot of fitness as a function of phenotype is asymmetrical (i.e. fitness drops off more quickly in one direction than in the other when we move away from an optimum phenotype). It is also different from asymmetry in the distribution of breeding values, which has long been known to influence evolution [43], as well as the asymmetry in the expected change under selection that appears in some diffusion models [44]. Rather, the odd-moment effect is a directional evolutionary force that appears when different individuals have different degrees of asymmetry in their fitness distributions.

### Associations between offspring number and offspring phenotype

Equation 2 contains two terms representing covariance between the degree to which offspring differ from their parents (*δ*) and contribution to population growth ($\hat{\Omega }$). The first of these, cov($\hat{\delta },\hat{\Omega }$), captures the degree to which the individuals that have the highest expected contribution to population growth are also those that produce offspring that deviate most from their parents. In this term, the covariance is over the entire population, and may result either from a direct causal influence of fitness on offspring phenotype, or any fortuitous association in which the phenotype that confers the highest value of $\hat{\Omega }$ happens to also be associated with individuals who's offspring differ most (or least) from their parents.

By contrast, the term cov_*i*_(*δ*, *Ω*) measures the covariance within an individual between *w *and *δ*, meaning that if that individual produces more offspring than expected, then its offspring's phenotypes are expected to deviate more (or less) from its own (In Equations 1 and 2, this property of individuals is averaged over the entire population). This term will be nonzero when there is a direct connection between how many offspring an individual produces and the phenotypes of those offspring. One example of this would be a case in which, for any given individual, producing more offspring directly causes those offspring to be smaller. Such "offspring-size/clutch-size tradeoffs" [45] are expected in cases in which parents provision their offspring with limited resources, so producing more offspring necessitates giving fewer resources to each one. This term would also be nonzero in cases in which the offspring of a particular individual interact with one another in such a way that their development is influenced by how many siblings they have (this will include in-utero interactions).

### Relation between selection and directional stochastic evolution

#### Definition of selection

As mentioned above, I am defining selection as differential expected production of descendants that is causally influenced by variation in phenotype. Under this definition, selection is captured by the term cov(*ϕ*, $\hat{w}$), assuming that the association is due to causal impacts of *ϕ *on $\hat{w}$. Some researchers (and reviewers) define selection differently, as any process involving differential survival or reproduction that leads to a predictable change in allele frequency [33]. This definition runs into problems with processes like balancing selection, that do not lead to any directional change.

Furthermore, defining selection as everything that leads to directional change effectively precludes it from being a specific evolutionary mechanism, since it is defined as the set of all mechanisms that produce a particular result. Defining selection in this way makes it effectively synonymous with directional evolution.

By contrast, if we define selection as differential production of descendants (or differential survival and reproduction) that is causally determined by variation in phenotype, then we have identified a particular class of mechanisms that will produce predictably different consequences under different conditions. Balancing and stabilizing selection are easily accommodated by this definition.

These definitional issues have no bearing on the evolutionary importance of the processes discussed above. Readers who prefer to define selection as anything that produces directional change may read the following section as a discussion of different components of selection.

#### Directional stochastic effects

**Figure 4:**
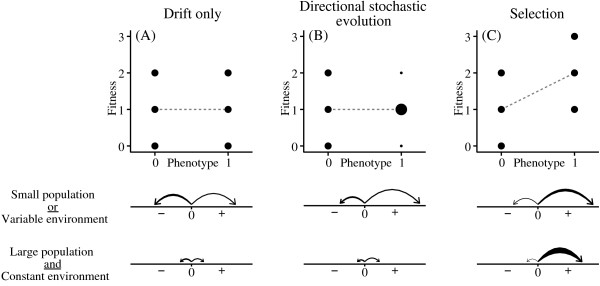
**Schematic illustration of different evolutionary processes**. Examples of fitness distributions corresponding to different processes. The variation in fitness for each phenotype is due to pure demographic stochasticity. The dashed gray lines show the regression of expected fitness ($\hat{w}$) on phenotype. Below each fitness distribution are diagrams illustrating both the probability of the trait increasing or decreasing (indicated by the thickness of each arrow) and the magnitude of change in each direction (indicated by the length of each arrow).

The even- and odd-moment effects discussed above result from the same random variation in individual reproduction that causes drift. To understand the relationship between directional stochastic evolution, drift, and selection, it is important to distinguish between two different factors that can produce directional change: 1) the relative probabilities of the mean phenotype increasing or decreasing, and 2) the expected magnitude of change in each direction (Fig. 4). In the case of pure drift, these factors exactly cancel one another out – a higher probability of moving in one direction is exactly balanced by a larger step size in the other direction – leading to a net expected change of zero (in some special cases, such as two alleles at equal frequency, both the probability and step size are the same in both directions). Drift is thus non-directional (E(Δ$\bar{\phi }$) = 0), but has a magnitude measured by the variance in Δ$\bar{\phi }$. Drift can occur only if there is variation in the fitness distributions of individuals. As population size increases, the magnitude of drift decreases, approaching zero as *N *→ ∞. (In Fig. 4, all fitness variation results from pure demographic stochasticity. If fitness variation results from environmental variation, then there can be directional change even in cases like that in Fig. 4A.)

Directional stochastic effects behave like drift insomuch as they require that individuals have distributions of possible fitness values. However, the probability of moving in each direction and the expected step size in each direction do not cancel one another out. In a constant environment, the expected magnitude of change declines towards zero as *N *→ ∞, as in the case of drift.

In the case of selection, there is both a higher probability of the mean phenotype changing in one direction and a larger expected step size in that direction. Unlike drift and directional stochastic evolution, selection can take place even if there is no variation in any of the individual fitness distributions. As population size increases, the expected change due to selection decreases somewhat, but does not go to zero; instead asymptotically approaching the value $\frac{\mathrm{cov}(\phi ,\hat{w})}{\hat{\bar{w}}}$. Furthermore, the probability of the population changing by this amount in the direction specified by selection approaches 1 as population size approaches infinity.

Selection also differs from the directional stochastic terms in that it involves covariance between phenotype and the first raw (not central) moment of the fitness distribution. By contrast, all of the directional stochastic terms involve central moments of *w*. Also, the denominator in the first (selection) term is the harmonic mean of $\bar{w}$, (H($\bar{w}$)), whereas all subsequent terms involve dividing by powers of the expected value of $\bar{w}$, ($\hat{\bar{w}}$).

### Consequences for adaptive landscape models

The concept a surface describing fitness as a function of a set of phenotypic traits (one version of the "adaptive landscape"), has a long history in evolutionary theory [46,47], and variants of this idea have recently been presented as unifying concepts in evolutionary biology [48,49]. This is indeed an important kind of abstraction that both hones our intuition about evolution and allows us to visualize an important set of formal evolutionary models. The results presented above, though, show that thinking of evolution in terms of an adaptive landscape can also lead us to miss important evolutionary processes.

By its nature, an adaptive landscape treats $\bar{w}$ as a number, rather than as a random variable (which has a distribution, rather than a single value). Because of this, both the amplification of selection differentials and all directional stochastic evolutionary processes are eliminated from adaptive landscape models. Even in a stable environment with frequency independent selection, directional stochastic effects could pull a population downhill on an adaptive landscape.

One possible way around this would be to to consider a surface of expected relative fitness, essentially plotting $\hat{\Omega }$, rather than $\hat{w}$ or $\hat{\bar{w}}$, as a function of phenotype [10,32]. However, Equation 3 shows that $\hat{\Omega }$ is itself a function of population size, meaning that such a landscape would change shape as *N *changes even if selection is not density dependent in the classical sense (meaning that the fitness distribution of each individual is independent of *N*).

The more appropriate visual image would be an adaptive fog, with variable density and thickness corresponding to different fitness distributions for different phenotypes. The dynamics of evolution through such a fog are described by Equations 1 and 2, and are determined not only by the slope of expected mean fitness ($\bar{w}$) but also by variations in the thickness of the fog and by population size (since this will influence H($\bar{w}$)). Unfortunately, this image lacks the visual simplicity of the adaptive landscape, which remains a very useful concept but should be recognized as an approximation based on the assumption that fitness values are fixed.

### Relation between Equation 1 and the Price equation

I refer to Equation 1 (and 2, which is equivalent) as a stochastic version of the Price equation because it is derived in an analogous way. Equations 1 and 2 are not, however, equivalent to the Price equation and can not be derived directly from it (specifically, the term $\bar{{\mathrm{cov}}_{i}(\delta ,\Omega )}$ can not be derived simply by treating *w *and *δ *as random variables in the Price equation). The reason for this is that the Price equation is derived by treating fitness and offspring phenotype as parameters, having numerical values, rather than as random variables, which have distributions. This is why the Price equation is exact only in hindsight, when we know how many descendants each individual had and what their phenotypes are. (Graffen [24] derived an equation equivalent to 2 under the assumption that *δ *= 0).

We can, of course, apply the Price equation to looking forward in time if we are willing to assume that expected fitness ($\hat{w}$) can be used in place of the actual number of descendants that an individual will leave, and to further assume that we can predict the phenotypes of offspring. (Price himself appears to make this assumption in his example of students with different IQs taking a course [12]). However, the preceding discussion shows that considering only expected fitness (instead of $\hat{\Omega }$) leads us to miss an entire class of evolutionary mechanisms.

How, then, is it possible for both Equations 1 and the Price equation to be exactly true given that they are different? Any evolving system must satisfy both Equation 1 and the Price equation. However, if we focus on change over a particular generation, these equations are appropriate at different times. Prior to reproduction, when fitness and offspring phenotype are not yet exactly determined, Equations 1 and 2 are exact descriptors of the expected change over the coming generation. After reproduction has taken place, the Price equation will, retrospectively, be an exact description of what just transpired.

### The limitations of general theories in biology

Equations 1 and 2 and Price's equation are general in the sense that they apply exactly to any evolving system. Note, though, that this does not mean that they answer all of our questions about evolution. Two objections that are sometimes raised about the Price equation (and which apply to Equations 1 and 2 as well) are that it is not dynamically sufficient [16], and that it does not directly address some important evolutionary questions, such as the probability of fixation of an allele.

As discussed in the Methods section (see "Worked example"), whether or not Equation 1 can be iterated into the future (*i.e*. is dynamically sufficient) is determined by the kinds of phenotypes that we are studying and what assumptions we make about them (see also [50]). In the case of a population containing two distinct phenotypes (such as a one locus haploid model with two alleles), the entire distribution is uniquely defined by the mean. In such a case, we can iterate Equation 1 through time with no further simplifying assumptions. If there are more than two phenotypes (such as in diploid models where genotypes take the role of *ϕ*), then some further assumption, such as Hardy-Weinberg equilibrium, is necessary to achieve dynamic sufficiency. In the case of a continuous phenotypic trait, a simple way to make the model dynamically sufficient is to assume that the trait is normally distributed, meaning that we need only calculate the change in the mean and variance (change in variance is obtained from Equations 1 or 2 by substituting (*ϕ *- $\bar{\phi }$)^2 ^for *ϕ *[13,51]).

These are exactly the same assumptions that make models in population and quantitative genetics dynamically sufficient. Thus, the general equations discussed here are no less dynamically sufficient than any of the standard models (since these are special cases). The general equations simply apply to a much broader set of cases, some of which do not allow for a single, compact, dynamically sufficient equation [52].

Another criticism is that these equations describe only the change over a generation, which does not, by itself, answer some evolutionary questions. However, the change in mean phenotype (of which a special case is change in the frequency of an allele or strategy) is one of the most basic pieces of formal evolutionary theory. In some fields, such as quantitative genetics, change in $\bar{\phi }$ is the primary quantity of interest. In other cases, such as evolutionary game theory, it is a key factor in evaluating the quantity of interest (evolutionary stability). In population genetics, change over a generation is sometimes the quantity of interest, and even when it is not (such as when the goal is to calculate fixation probabilities), change in allele frequency is an essential part of the answer (*e.g*. it defines *M *(*p*) in a diffusion equation). Though the general models discussed here do not answer all of our questions, their value lies in their ability to generalize and unify special case models, and to give us insights into the mechanics of evolution that can be obscured by the assumptions necessary to predict the long term behavior of particular model systems.

## Conclusion

The interplay of deterministic and stochastic processes is central to much of evolutionary theory. Unfortunately, our most general mathematical description of evolution, the Price equation, is not well suited to the study of stochasticity. This is because the Price equation describes evolution exactly only after change has taken place, meaning that it contains no stochastic terms (since all parameters are known exactly in hindsight). A general stochastic evolution equation, derived in a similar way to the Price equation but different in that fitness and offspring phenotype are treated as random variables, reveals a number of general rules about the interaction of deterministic and stochastic processes in evolution.

One result is that variation in mean population fitness, resulting either from small population size or environmental fluctuations, tends to amplify the effects of selection. This suggests that the adaptive potential of small populations may be greater than has been assumed. Another result is that the well known tendency for populations to be pulled towards phenotypes with minimum variance in fitness turns out to be a special case of a general trend to minimize symmetric variation in fitness. This process can actually cause variance in fitness to increase, so long as higher even moments decrease. This even-moment effect is matched by an odd-moment effect, which tends to pull populations towards phenotypes with maximum positive asymmetry in fitness.

Both the even- and odd-moment effects can drive a population to evolve towards phenotypes with lower expected fitness. This is consistent with (and is a generalization of) previous results showing that differential variance in fitness can drive directional evolution. It is not, however, consistent with the idea that geometric mean fitness determines the direction of evolution. Instead, in cases of perfect heritability, the direction of evolution is determined by the expected value of individual fitness divided by mean population fitness ($\frac{w}{\bar{w}}$), conditional on the population not going extinct. This confirms the importance of "expected relative fitness" [10,32], when defined properly, as a determining factor in evolutionary dynamics.

Finally, the general equations presented here contain a term capturing the direct influence of parental fitness on offspring phenotype. This term, which has no analog in the Price equation, may be important in the many cases in which parents provision their offspring or in which individual development is influenced by interactions with siblings. This also illustrates the value of treating offspring phenotype, like fitness, as a random variable.

## Methods

### Derivation of Equation 1

In the following derivations, it is essential to distinguish between: 1) the expected value of a random variable and 2) the average value of that variable in a population. For example: before reproduction takes place, individual fitness (*w*) is a random variable, meaning that each individual has a distribution of possible fitness values. The expected value of this distribution, for a particular individual, is $\hat{w}$. It is critical to distinguish between this expected value and the average value of *w *in the population, denoted $\bar{w}$, which is an important term in its own right (it measures per capita population growth rate). $\bar{w}$ is itself a random variable, since prior to reproduction we can not know exactly how the population will change in size. We thus have $\hat{\bar{w}}$ as the expected value of average fitness. An important identity is E(Ave(*x*)) = Ave(E(*x*)) or $\hat{\bar{x}}=\bar{\hat{x}}$ (this is easily shown by noting that $\text{E}(\frac{1}{N}\sum_{i=1}^{N}x_{i})=\frac{1}{N}\sum_{i=1}^{N}\text{E}(x_{i})$).

Define ${\bar{\phi }}^{\prime }$ as the mean phenotype in the population after one time interval, and $\phi_{ij}^{o}$ as the phenotype of the *j*^*th *^descendant of individual *i *in the current population. Then, conditional on $\sum_{i=1}^{N}w_{i}\neq 0$:

(6) $$\text{E}({\bar{\phi }}^{\prime })=\text{E}\left(\frac{\sum_{i=1}^{N}\sum_{j=1}^{w_{i}}\phi_{ij}^{o}}{\sum_{i=1}^{N}w_{i}}\right)$$

If we denote the average phenotype of descendants of individual *i *as simply $\phi_{i}^{o}$, then $\sum_{j=1}^{w_{i}}\phi_{ij}^{o}=\phi_{i}^{o}w_{i}$ and $\sum_{i=1}^{N}w_{i}=N\bar{w}$, and Equation 6 becomes:

(7) $$\text{E}({\bar{\phi }}^{\prime })=\text{E}\left[\text{Ave}\left(\frac{\phi^{o}w}{\bar{w}}\right)\right]=\hat{\bar{\left(\frac{\phi^{o}w}{\bar{w}}\right)}}$$

Using the fact that E(Ave(*x*)) = Ave(E(*x*)) and noting that the rule E(*xy*) = cov(*x, y*) + E(*x*)E(*y*) applies as well to Ave(), we can expand Equation 7 to yield:

(8) $$\text{E}({\bar{\phi }}^{\prime })=\bar{\mathrm{cov}\left(\phi^{o},\frac{w}{\bar{w}}\right)}+\mathrm{cov}\left[{\hat{\phi }}^{o},\hat{\left(\frac{w}{\bar{w}}\right)}\right]+\hat{{\bar{\phi }}^{o}}\hat{\bar{\left(\frac{w}{\bar{w}}\right)}}$$

Defining $\hat{\bar{\delta }}=\hat{\bar{\phi^{o}}}-\bar{\phi }$, noting that Ave[E(*w*/$\bar{w}$)] = E[Ave(*w*/$\bar{w}$)] = 1, and using the fact that $\text{E}(\Delta \bar{\phi })=\text{E}({\bar{\phi }}^{\prime })-\bar{\phi }$, we get:

(9) $$\text{E}(\Delta \bar{\phi })=\mathrm{cov}\left[{\hat{\phi }}^{o},\hat{\left(\frac{w}{\bar{w}}\right)}\right]+\bar{\mathrm{cov}\left(\phi^{o},\frac{w}{\bar{w}}\right)}+\hat{\bar{\delta }}$$

Defining $\Omega_{k}=\left(\frac{w_{k}}{\bar{w}}|\bar{w}\neq 0\right)$ and noting that $\phi_{i}^{o}$ = *ϕ*_*i *_+ *δ*_*i *_yields Equation 1.

### Derivation of Equation 3 and 4

For a random variable, *x*, denote the difference between *x *and its expected value as x*; so *x *= E(*x*) + x*, E(x*) = 0, and E[(x*)^*n*^] is the *n*^*th *^central moment of *x *(this is just the delta method). We can now write ${\hat{\Omega }}_{k}$ as:

(10) $${\hat{\Omega }}_{k}=\frac{\text{E}(w_{k})}{\text{E}(\bar{w})}\text{E}\left[\left(1+\frac{w_{k}^{*}}{E(w_{k})}\right){\left(1+\frac{{\bar{w}}^{*}}{\text{E}(\bar{w})}\right)}^{-1}\right]$$

The Taylor series expansion of Equation 10 does converge (so long as we calculate all probabilities conditional on $\bar{w}$ ≠ 0), but it contains a rather non intuitive mix of terms involving both *w *and $\bar{w}$, producing a mix of higher moments that is difficult to interpret biologically. We can make things clearer by noting that Equation 10 involves the sum of two different series. One of these contains terms involving the mixed moments of *w *and $\bar{w}$, while the other contains only moments of $\bar{w}$. This second series can be pulled out by noting that it is the reciprocal of the harmonic mean of $\bar{w}$:

(11) $$\frac{1}{\text{H}(\bar{w})}\equiv \text{E}\left(\frac{1}{\bar{w}}\right)=\frac{1}{\text{E}(\bar{w})}\text{E}\left[{\left(1+\frac{{\bar{w}}^{*}}{\text{E}(\bar{w})}\right)}^{-1}\right]$$

Combining Equations 10 and 11 yields:

(12) $${\hat{\Omega }}_{k}=\frac{\text{E}(w_{k})}{\text{H}(\bar{w})}+\frac{\text{E}(w_{k})}{\text{E}(\bar{w})}\text{E}\left[\frac{w_{k}^{*}}{\text{E}(w_{k})}{\left(1+\frac{{\bar{w}}^{*}}{\text{E}(\bar{w})}\right)}^{-1}\right]$$

Expanding ${\left(1+\frac{{\bar{w}}^{*}}{\text{E}(\bar{w})}\right)}^{-1}$ in a Taylor series and taking the expected value yields Equation 3:

(13) $${\hat{\Omega }}_{k}=\frac{{\hat{w}}_{k}}{\text{H}(\bar{w})}+\sum_{i=1}^{\infty }\frac{{\left(-1\right)}^{i}\mu_{i+1}(w_{k}{\bar{w}}^{i})}{{\hat{\bar{w}}}^{i+1}}$$

Where μ_*i*+1_(*w*_*k *_${\bar{w}}^{i}$) is the (*i *+ 1)^*st *^mixed central moment of *w*_*k *_and ${\bar{w}}^{i}$. If we assume that H($\bar{w}$) = $\hat{\bar{w}}$ and consider only the first term in the summation, then Equation 13 yields Lande's expected relative fitness [32] (since μ_2_(*w*_*k *_$\bar{w}$) = cov(*w*_*k*_, $\bar{w}$)). Proulx [9,34,53] has presented a series that groups terms differently than does Equation 13, grouping them based on their order in an approximation of small variance in offspring numbers.

If the actual number of descendants of different individuals are independent – meaning that the number of descendants of individual *k *is independent of whether individual *j *leaves more or fewer descendants than expected – then cov(*w*_*k*_, *w*_*j *≠ *k*_) = 0, so $\mu_{2}(w_{k}\bar{w})=\frac{1}{N}\mathrm{var}(w_{k})$ and $\mu_{3}(w_{k},{\bar{w}}^{2})=\frac{1}{N^{2}}\mu_{3}(w_{i})$. We thus have:

(14) $${\hat{\Omega }}_{k}=\frac{{\hat{w}}_{k}}{\text{H}(\bar{w})}-\frac{\mathrm{var}(w_{k})}{N{\hat{\bar{w}}}^{2}}+\frac{\mu_{3}(w_{k})}{N^{2}{\hat{\bar{w}}}^{3}}-\cdots$$

Substituting Equation 14 into cov(*ϕ*, $\hat{\Omega }$) yields Equation 4.

### Derivation of Equation 5

Consider a case in which individuals with certain phenotypes are consistently influenced the same way by environmental variation across generations (*e.g*. wet and dry years occur at random, and wet years influence the fitness of large individuals differently from the way that they influence small individuals). In such a case, we can write individual fitness as *w*_*i *_= ${\tilde{w}}_{i}$ + *s*_*i*_, where ${\tilde{w}}_{i}$ is the expected fitness, in the current environment, of individuals with the same phenotype as individual *i*, and *s*_*i *_is the deviation from this expected fitness due to pure demographic stochasticity. In this case,

(15) $$\mu_{2}(w_{i}\bar{w})=f_{\phi_{i}}\mathrm{var}({\tilde{w}}_{k})+2f_{\phi_{i}}\mathrm{cov}(s_{i},{\tilde{w}}_{i})+\frac{\mathrm{var}(s)}{N}$$

If the effects of pure demographic stochasticity are independent of the environment, and *N *is large, then we need only consider the term $f_{\phi_{i}}$ var(${\tilde{w}}_{i}$).

Under the same assumptions, the third moment effect is captured by:

(16) $$\mu_{3}(w_{i}{\bar{w}}^{2})\approx f_{\phi_{i}}^{2}\mu_{3}({\tilde{w}}_{i})$$

Substituting $\mu_{2}(w_{i}\bar{w})=f_{\phi_{i}}\mathrm{var}({\tilde{w}}_{i})$ and $\mu_{3}(w_{i}{\bar{w}}^{2})=f_{\phi_{i}}^{2}\mu_{3}({\tilde{w}}_{i})$ into Equation 3 yields Equation 5.

### Worked Example

**Figure 5:**
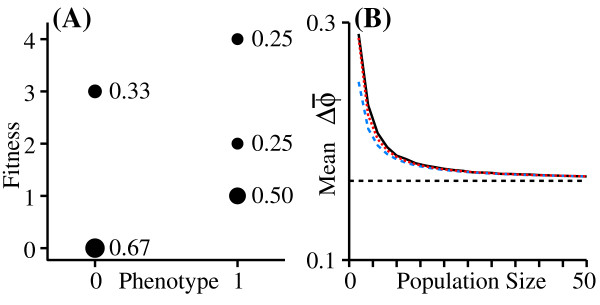
**Approximating the curve of **$\hat{\Delta \bar{\phi }}$**using moments**. A fitness distribution (A) and the corresponding expected change in mean phenotype for different population sizes (B). The solid black curve in (B) is the curve resulting from 100,000 monte-carlo runs per value of *N*. The dashed blue line in (B) shows the result of using Equation 17 and considering only the first two terms in the expansion (up to and including var($\bar{w}$)). The dashed red line shows the result of using the first four terms of the expansion (up to and including μ_4_($\bar{w}$)).

Figure 5 shows a case in which directional selection is acting simultaneously with the even- and odd-moment effects. In order to analytically solve for the selection differential as a function of population size, we need to calculate $\hat{\Omega }$. The most difficult term in Equation 3 to calculate is the first one, containing the reciprocal of the harmonic mean of $\bar{w}$. For very small populations, we can sometimes calculate 1/H($\bar{w}$) directly. For larger populations, though, we need to use a series approximation. Expanding the right-hand side of Equation 11 yields:

(17) $$\begin{array}{lll} \frac{1}{\text{H}(\bar{w})} & = & \frac{1}{\hat{\bar{w}}}+\frac{\mathrm{var}(\bar{w})}{{\hat{\bar{w}}}^{3}}-\frac{\mu_{3}(\bar{w})}{{\hat{\bar{w}}}^{4}}+\frac{\mu_{4}(\bar{w})}{{\hat{\bar{w}}}^{5}}-\cdots \\ & = & \frac{1}{\hat{\bar{w}}}+\sum_{i=2}^{\infty }\frac{{\left(-1\right)}^{i}\mu_{i}(\bar{w})}{{\hat{\bar{w}}}^{i+1}} \end{array}$$

Next, we need to calculate the moments of $\bar{w}$ from the moments of the fitness distributions associated with each phenotype (which is what we are starting out with). If the actual fitness of each individual is independent of that of others in the same generation, then for the case in which there are *P *distinct phenotypes and *n*_*i *_individuals with phenotype *i*, the second, third, and fourth central moments of $\bar{w}$ are given by:

(18) $$\mathrm{var}(\bar{w})=\sum_{i=1}^{P}\frac{n_{i}}{N^{2}}\mathrm{var}(w_{i})$$

(19) $$\mu_{3}(\bar{w})=\sum_{i=1}^{P}\frac{n_{i}}{N^{3}}\mu_{3}(w_{i})$$

(20) $$\begin{array}{ll} \mu_{4}(\bar{w}) & =\\ & \sum_{i=1}^{P}\left(\frac{n_{i}}{N^{4}}\mu_{4}(w_{i})+3\frac{n_{i}(n_{i}-1)}{N^{4}}\mathrm{var}{\left(w_{i}\right)}^{2}\right)\\ & +3\sum_{i=1}^{P}\sum_{j\neq i}\frac{n_{i}n_{j}}{N^{4}}\mathrm{var}(w_{i})\mathrm{var}(w_{j}) \end{array}$$

Equation 20 is derived using the fact that $\bar{w}$ is a sum of different values, and assuming that the fitness values of different individuals are independent. The equations for the higher moments get large, but have a straightforward form. The number of terms in the series in Equation 17 that are needed to get a good approximation is determined by the individual fitness distributions and population size. Figure 5 shows an example in which using only the first two terms yields an underestimate for small populations, but using the first four terms yields a good fit at all population sizes. In the example in Figure 5, there are two phenotypes, scored as 0 and 1, with ${\hat{w}}_{0}$ = 1, ${\hat{w}}_{1}$ = 2, var(*w*_0_) = 2, var(*w*_1_) = 1.5, μ_3_(*w*_0_) = 2, μ_3_(*w*_1_) = 1.5, μ_4_(*w*_0_) = 6, μ_4_(*w*_1_) = 4.5.

Next, we need to specify the current frequencies of each phenotype and solve for the covariance terms. For the case of only two phenotypes, assigned values 0 and 1 and having frequencies *f*_0 _and *f*_1_, the general rule is:

(21) cov(*ϕ*, μ_*i*_(*w*)) = *f*_0_*f*_1 _[μ_*i*_(*w*_1_) - μ_*i*_(*w*_0_)]

For this example, I set the frequencies to be equal, so that *n*_0 _= *n*_1 _= *N/*2. For this case, we have $\hat{\bar{w}}$ = 1.5, cov(*ϕ*, $\hat{w}$) = 0.25, cov(*ϕ*, var(*w*)) = -0.125, cov(*ϕ*, μ_3_(*w*)) = -0.125, cov(*ϕ*, μ_4_(*w*)) = -0.375, The dashed curves in the figure were derived by using the moments of the fitness distributions for each phenotype to approximate 1/H($\bar{w}$) (using Equations 18 – 20 and Equation 17) and to calculate the covariance terms using Equation 21.

Note that, for the case of two phenotypes, we can calculate all of the necessary terms using only the fitness distributions for each phenotype and the mean phenotype (from which we can calculate the phenotypic frequencies if there are only two). We can thus iterate this process forward in time. If there are more than two phenotypes, then iteration is not possible unless we make further assumptions (such as assuming Hardy-Weinberg frequencies for genotypes or a normal distribution with fixed variance for a continuous trait), that allow us to specify the entire distribution given only the mean.

It is sometimes necessary to use moments higher than μ_4_($\bar{w}$) for very small populations with highly asymmetrical fitness distributions. As Equations 18 – 20 show, though, the higher moments of $\bar{w}$ contain increasing powers of $\frac{1}{N}$. Using only the first few terms on the right-hand side of Equation 17 thus tends to give a very good approximation for populations larger than a few dozen individuals.

### Monte-carlo simulations

The monte-carlo simulations used asexual individuals with non overlapping generations. The value of Δ$\bar{\phi }$ was calculated by looking over a single generation starting with *N *individuals, evenly divided between the two phenotypic values (so initially $\bar{\phi }$ = 0.5). Each individual's contribution to the next generation is drawn at random from its fitness distribution and the new mean phenotype is calculated. The curves presented are the averages of 100,000 runs for each population size.

## Authors' contributions

SHR did the work and wrote the paper.
